# Image Quality Evaluation of a Digital Radiography System Made in Thailand

**DOI:** 10.1155/2021/3102673

**Published:** 2021-11-16

**Authors:** Udomchai Techavipoo, Nattawut Sinsuebphon, Sakunrat Prompalit, Saowapak Thongvigitmanee, Walita Narkbuakaew, Atthasak Kiang-ia, Tanapon Srivongsa, Pairash Thajchayapong, Utairat Chaumrattanakul

**Affiliations:** ^1^Assistive Technology and Medical Devices Research Center, National Science and Technology Development Agency, Pathum Thani 12120, Thailand; ^2^Department of Radiology, Faculty of Medicine, Thammasat University, Pathum Thani 12120, Thailand

## Abstract

**Background:**

The National Science and Technology Development Agency (NSTDA) in Thailand researched and prototyped digital radiography systems under the brand name BodiiRay aiming for sustainable development and affordability of medical imaging technology. The image restoration and enhancement were implemented for the systems.

**Purpose:**

The image quality of the systems was evaluated using images from phantoms and from healthy volunteers.

**Methods:**

The survey phantom images from BodiiRay and other two commercial systems using the exposure settings for the chest, the abdomen, and the extremity were evaluated by three experience observers in terms of the high-contrast image resolution, the low-contrast image detectability, and the grayscale differentiation. The volunteer images of the chests, the abdomens, and the extremities from BodiiRay were evaluated by three specialized radiologists based on visual grading on 5-point scaled questionnaires for the anatomy visibility, the image quality satisfaction, and the diagnosis confidence in using the images.

**Results:**

BodiiRay phantom results were similar to those from the commercial systems. The overall performance averaged across the exposure settings showed that BodiiRay was slightly better than Fujifilm FDR Go in the low-contrast detectability (*p* = 0.033) and in the grayscale differentiation (*p* = 0.004). It was also slightly better than Siemens YSIO Max in the high-contrast resolution (*p* = 0.018). The images of chest, pelvis, and hand phantoms illustrated comparable visual quality. For volunteer images, the percentage of the images scored ≥4 ranged from 61% to 99%, 23% to 92%, and 96% to 99% for the chest, abdomen, and extremity images, respectively. The average score ranged from 3.63 to 4.46, 3.18 to 4.21, and 4.41 to 4.51 for the chest, abdomen, and extremity images, respectively.

**Conclusion:**

The phantom image results showed the comparability of these systems. The clinical evaluation showed BodiiRay images provided sufficient image qualities for digital radiography of these body parts.

## 1. Introduction

Digital radiography (DR) has become an essential medical imaging modality for diagnosis. It has replaced the traditional photographic films in the screen-film radiography, as well as phosphor screens housed in cassettes in the computed radiography (CR) [1–3]. DR systems are composed of two parts: (1) an X-ray source that contains a high-voltage and high-frequency electrical generator and an X-ray tube to produce X-ray beams and (2) an X-ray detector or a flat panel detector (FPD) that converts X-ray photons into electrical charges by a matrix of photodiodes and readout into a raw image data matrix. DR has many advantages, e.g., instant image and image preview, special image enhancement availability, removals of costly film or CR processing steps, a wider dynamic range, and lower patient radiation dose. However, the cost of the DR systems is still expensive for the hospitals in the rural area of the low- and middle-income countries.

The Assistive Technology and Medical Devices Research Center (A-MED) and the National Electronics and Computer Technology Center (NECTEC) under the National Science and Technology Development Agency (NSTDA) aimed for a sustainable development of medical imaging technology and made it affordable for people in the rural area in order to provide better medical care. Medical Imaging System Research Team (MIS) in A-MED researched and prototyped many DR systems, e.g., a U-Arm type [4], a stand type for the chest X-ray, a retrofit type for upgrading the existing film/CR systems to DR, and a mobile type. These DR systems were produced under a brand name “BodiiRay.” These BodiiRay systems were proposed in order to serve the government policy of import reduction and local business promotion. Almost all of the DR systems that were currently available in the market and were used in the hospitals in Thailand were imported. Therefore, the country spent an alarming high budget for the imports. To response to this situation, the government introduced a policy for the public hospitals to purchase the products that were researched and innovated by Thai institutes. These products needed to meet the international standards and to be comparable to the imported products.

BodiiRay systems were designed to fit local medical applications. For example, the stand type, so called “BodiiRay S,” comprised two stands (one for installing an X-ray source and the other for an X-ray detector) with synchronous patient's height adjustability. It was suitable for chest X-ray in a routine medical examination. The retrofit type, so called “BodiiRay R,” comprised a wireless X-ray detector and a computer. It reduced the cost of upgrading existing films/CR systems by replacing old X-ray receptors but still reusing the old X-ray sources. The mobile type, so called “BodiiRay M,” comprised a wireless X-ray detector, a computer, and an X-ray source installed on a portable cart. It was suitable for patients with a difficulty for transfer to an X-ray room.

In-house software running inside BodiiRay systems was composed of three main parts as follows. (1) The main program communicated with the other parts and with hospital databases, including the patient information database and the picture archiving and communication system (PACS). The main program also converted the final image into a format of the digital imaging and communications in medicine (DICOM), which was a required standard format for the PACS. (2) The acquisition program communicated with the hardware components, e.g., the FPD and the X-ray source. It also contained preprocessing software, i.e., bad pixel, offset, and gain corrections [5, 6], as well as the image restoration and enhancement [7, 8]. These converted the raw image data into a presentable image. (3) The image viewer displayed presentable images and communicated interactively with users. Postprocessing, such as image contrast and size adjustment, annotation insertion, and image transformations, was also contained in this part.

The image restoration and enhancement were implemented to improve the image quality. The nature of DR image quality was affected by the modulation transfer function (MTF) and the signal to noise ratio (SNR) performance of the FPD. The MTF described the spatial resolution of the imaging system as a function of the spatial frequency. In general, the higher spatial frequency, the lower its magnitude. The SNR performance was related to the number of incident photons on the FPD and the efficiency to convert them into signal. The number of incident photons varied spatially based on the attenuation of the anatomy that the X-ray passed. For these reasons, the restoration could be done using the inverse filter derived from the MTF to recover the attenuated spatial frequency. However, it needed to be spatially adaptive [9] to compensate for the noise. The image contrast was based on the attenuation differences of the anatomies in superposition. Poor image contrast occurred at the area of high attenuation. Many contrast enhancement algorithms, e.g., unsharp masking [10, 11], histogram equalization (HE) [12, 13], and multiscale image processing [13], were investigated. The HE divided an image into small tiles and equalized their histograms using their inverse cumulative density functions to map the pixel values. Bilinear interpolation of the maps was also applied to reduce the blocking artifact. This had been used moderately in our first version [4] to avoid blocking artifact and unrealistic impression. In the current version, the image enhancement was based on multiscale image processing. It was developed and tested positively in a pilot study [8].

In the development of this DR software, the images from different kinds of phantoms were evaluated to verify the software performance and to adjust its image restoration and enhancement algorithm. This step was done in collaborations with radiologists and radiologic technologists of multiple institutes to customize the software for user-friendly interface and for acceptable image quality. The image quality from the BodiiRay DR system was compared with those of other DR systems. Its software was also adjusted according to users' comments to meet their satisfaction.

In this article, the comparison of the phantom images of the BodiiRay DR system to those of other DR systems was done, and the image quality was reported. After this phantom image comparison, a clinical image evaluation on normal volunteers was performed. This clinical image evaluation was approved by the ethics committee, and the volunteers were informed consents before participation. The quality of each volunteer image was evaluated by radiologists using a manual scoring on a questionnaire. The statistics of these scores were reported.

## 2. Materials and Methods

### 2.1. DR Systems

A BodiiRay DR and two commercial DR systems were used in phantom images comparisons. These commercial DR systems was FDR Go (DR-ID-1211SE, Fujifilm, Japan) and Ysio Max (Wi-D, Siemens, Germany). The specifications of the FPD and the X-ray sources for these DR systems were described in Table 1. In general, the X-ray sources were 32 to 80 kW, providing voltage range between 40 and 150 kV and maximum current from 400 to 540 mA. The X-ray tube focal spots were varied from 0.6 to 1.5 mm. The X-ray detectors of these DR systems were all cesium iodide (CsI) scintillator with amorphous silicon (a-Si) thin film transistor (TFT) receptor type. Detector size are approximately 14 × 17 inches.

### 2.2. BodiiRay Image Enhancement

The enhancement was based on the multiscale image processing [8, 13]. The original image was deconstructed using low-pass filtering and downsampling. This step was done repeatedly for a certain number of iterations. In each iteration, the residual image was calculated by subtracting a low frequency image from the input image. This low frequency image was created by low-pass filtering the input, downsampling it, and then upsampling it back. The downsampling version of this was used as an input for the next iteration. This deconstruction provided a series of low frequency images, which was smaller and smoother, resembling a pyramid. It also provided a series of residual images resembling another pyramid as well.

An adaptive contrast adjustment was done on the residual images by remapping the pixel values for a better contrast. In addition, as each level of the image pyramids represented different spatial frequencies, it could be selectively enhanced for better detail and sharpness. After this adjustment, the reconstruction of the image was done by collapsing of the residual image pyramid. This step started from combining the topmost remapped residual image with its low frequency image and then upsampling. This process was continued until the final image was reconstructed.

The enhancement parameters were optimized based on the radiologist satisfaction on a pilot study, and the algorithm was optimized for computational performance. In the current version, the processing time was approximately 2 seconds for an image of 2476 × 3072 pixels on a computer with Intel® Core i7 9700 processor and 16 GB random access memory. The imaging time, i.e., from the exposure to the full image displayed on the monitor, was around 10 seconds.

### 2.3. Phantom Descriptions

#### 2.3.1. A Survey Phantom

**Figure 1:**
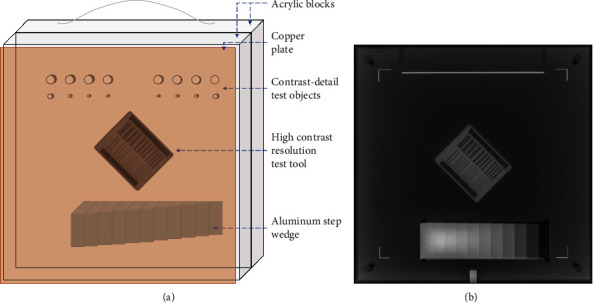
(a) A drawing of the radiographic survey phantom and (b) a sample of its X-ray image containing acrylic blocks, a copper plate, contrast-detail test objects, high contrast resolution test tool, and aluminum step wedge.

A radiographic survey phantom (170NJ, Gammex Inc., WI, USA) was used in the comparison. The drawing of this phantom and the sample of its X-ray image are shown in Figure 1. It was designed for three ranges of clinical settings by adjusting a copper plate with a different thickness located in front of the phantom, i.e., 2.0 mm for the chest, 2.4 mm for the abdomen, and no copper plate for the extremity. These copper plate thicknesses simulated the thicknesses of these body parts.

There were three groups of test objects imbedded inside an acrylic block. The descriptions of these test objects were as follows. (1) The high contrast resolution test tool contained line pairs of different measures ranged from 0.6 to 10 line pairs per millimeter (lp/mm). Observing higher lp/mm on an X-ray image implied a better image resolution. (2) The contrast-detail test objects were cylindrical holes aligned in two rows. The top row contained eight 0.375-inch-diameter holes with decreasing depths of 0.068, 0.049, 0.035, 0.025, 0.018, 0.013, 0.009, and 0.006 inch placing from the left to the right. The bottom row contained two sets of four 0.068-depth holes with decreasing diameters of 0.2, 0.15, 0.1, and 0.08 inch placing medially. On X-ray images, a hole with smaller depth or smaller diameter was more difficult to be distinguished from the background. Therefore, observing the smaller depth or smaller diameter implied better results on the lower contrast detectability. (3) The step wedge contained 11 aluminum steps, with an increment of a 0.125-inch step thickness. Observing more distinguished shades of the steps on an X-ray image implied higher grayscale levels of discrimination.

#### 2.3.2. Anthropomorphic Phantoms

**Figure 2:**
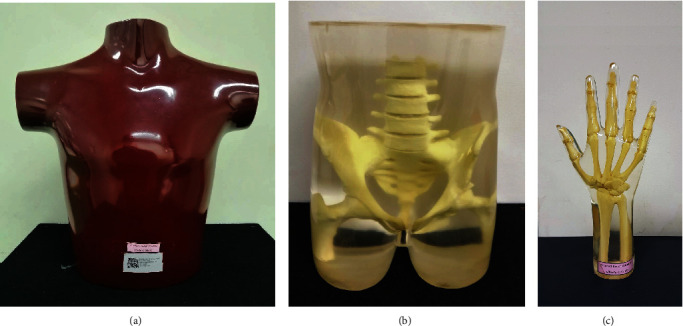
The anthropomorphic phantoms of (a) the chest, (b) the pelvis, and (c) the right hand used for visual assessment.

Three anthropomorphic phantoms, i.e., the chest, the pelvis, and the right hand (RS-315, RS-113T, and RS-114T, Radiology Support Devices Inc., CA, USA), were used for visual assessment. The chest phantom imitated the structure of the bones (e.g., spine, clavicle, scapula, sternum, and ribs) and soft tissues (lung, heart, coronary artery, and pulmonary arteries). In contrast, the pelvis and the hand phantoms contained only the structure of the bones. These phantoms were shown in Figure 2.

### 2.4. Phantom Image Comparison

The radiographic exposure settings for this phantom image comparison were generally used in hospitals for a portable X-ray machine. The source to image distance (SID) was set to 100 cm, and no grid was used. For the survey phantom, the copper plate thickness was selected to match the required settings of the chest, the abdomen, and the extremity. The radiographic exposure settings for the chest were 80 kV, 200 mA, and 2.5 mAs. Those for the abdomen were 80 kV, 160 mA, and 5.0 mAs. Those for the extremity were 53 kV, 160 mA, and 2.0 mAs, except that Siemens Ysio Max automatically set the exposure to 2.6 mAs for the chests, 5.1 mAs for the abdomen, and 2.1 mAs for the extremity. These settings were, respectively, used for the anthropomorphic phantoms of the chest, the pelvis, and the right hand.

The X-ray images for this comparison were generated using three DR systems, which were BodiiRay, Fujifilm FDR Go, and Siemens Ysio Max, and three radiographic exposure settings, which were for the chest, the abdomen, and the extremity, as described earlier. For each DR system, the survey phantom was adjusted the copper plate thickness to match with the procedure, and then, the radiographic exposure settings were set before an exposure. After that, the survey phantom was removed and replaced by the corresponding body section phantom before another exposure. The images in DICOM format were kept for analyzing in the next step.

Three experienced observers examined the survey phantom images, following a general guideline. This guideline allowed the observer to zoom and adjust the image contrasts but not to rotate the images. For the line pairs, they needed to be continuously separated at least half of the entire length to be accepted. For the contrast-detail depth, at least 50% of the cylindrical hole circumference needed to be distinct from the background to be accepted. For the contrast-detail diameter, the entire cylindrical hole needed to be distinct from the background to be accepted. For the aluminum step wedge, each step needed to be separated from the adjacent steps. The maximum separable lp/mm, the minimum distinguishable depth and diameter of the cylindrical holes, and the maximum number of separable steps of the step wedge were recorded. The statistic results, i.e., the mean and the standard deviation of the readings, were reported. The significance of the difference between the systems, analyzed using one-tailed paired *t*-test, was also reported. The generalized interobserver agreement *κ* [14] was also shown.

### 2.5. Volunteer Image Evaluation

This evaluation was performed on the X-ray images generated using the BodiiRay DR system. These images were from the healthy adult volunteers in three groups of 30 chest images, 30 abdomen images, and 30 extremity images. These volunteers were 24 males and 66 females, aged between 21 and 57 years with the average of 33 years (mean = 33.38, standard deviation = 8.73). Their body mass indexes (BMI) were between 17.4 and 33.2 with the average of 24.5. The radiographic exposure settings were based on those for the phantoms with some adjustments to match with patient size.

Three specialized radiologists performed visual grading analysis of the volunteer images. Each of them independently evaluated each image and scored the image on 5-point scaled questionnaires. The questions were on basic radiographic image quality assessments (i.e., the anatomy visibility and the satisfaction of the image display) and the confidence in using the image for diagnosis. The visibility of the lungs, the heart, the bronchi, the spine, the bone, and the bone trabecular pattern was for the chest images. The visibility of the air in the intestine, the soft tissues, and the fat planes under the skin and the density to differentiate the bone and fat were for the abdomen images. The visibility of the bone and the bone trabecular pattern was for the extremity images. The satisfaction of the image display was on the contrast/brightness, resolution, and the overall image display. The descriptions of the 5-point scale for the basic radiographic image quality assessment were 1 = invisible/unsatisfied, 2 = almost invisible/unsatisfied, 3 = 50 percent invisible/satisfied, 4 = acceptably visible/satisfied, and 5 = absolutely visible/satisfied. The descriptions of the 5-point scale for the radiologists' confidence were 1 = unconfident, 2 = slightly confident, 3 = moderately confident, 4 = high confident, and 5 = absolutely high confident.

There were 90 readings for each anatomy group, i.e., 30 images were read by 3 radiologists. After all images were scored, the scores were then analyzed on each question using the percentage of the readings that scored ≥4. This percentage implied the quality of the image for the basic radiographic image quality, which were acceptable to visualize the anatomy or satisfied by the radiologists on the image display. This percentage also implied high confidence for radiologists to use the images for diagnosis. The criteria to be recognized as a good image quality or radiologists' confidence were set to be as more than 80% of the readings that scored ≥4.

## 3. Results

### 3.1. Phantom Image Results

**Figure 3:**
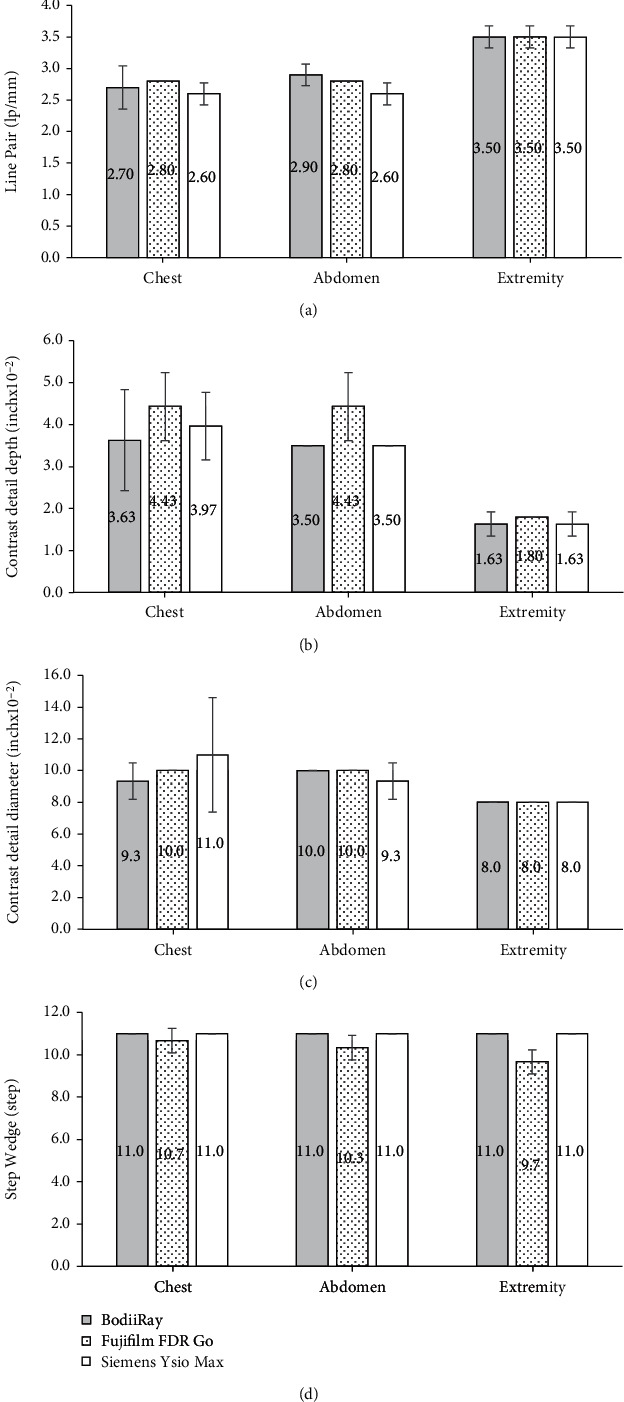
The survey phantom results averaged across the observers, (a) the maximum line pair per millimeter, (b) the minimum contrast detail depth, (c) the minimum contrast detail diameter, and (d) the maximum number of distinguished shades of the step wedge for the chest, the abdomen, and the extremity settings on the images of BodiiRay, Fujifilm FDR Go, and Siemens Ysio Max (using solid gray, dotted white, and solid white bars, respectively). The error bars were the standard deviations, and the numbers inside the bars were the means.

The results from the survey phantom for the maximum lp/mm, the minimum contrast detail depth, the minimum contrast detail diameter, and the maximum number of distinguished shades of the step wedge are shown in Figures 3(a)–3(d), respectively. These bar graphs showed the means and their standard deviations in error bars across three observers. Since the number of observers were small, the standard deviations in the error bars may not reflect the significance of the comparison. Some of the error bars were zero, meaning that all observers reported the same numbers. In general, the results from these DR systems were similar.

For the high contrast resolution results in lp/mm showed in Figure 3(a), the higher number of lp/mm, the better results. For the chest, Fujifilm FDR Go provided the average of 2.80 lp/mm, which is the slighter better than 2.70 lp/mm of BodiiRay and 2.60 lp/mm of Siemens Ysio Max. For the abdomen, BodiiRay provided the average of 2.90 lp/mm, which is slightly better than 2.80 lp/mm of Fujifilm FDR Go and 2.60 lp/mm of Siemens Ysio Max. For the extremity, all the machines were tied at the average of 3.50 lp/mm. Notice that the readings for the extremity setting were higher than those from the chest and the abdomen settings. This was from the lower scattering of the extremity setting because there was no copper plate installed in front of the survey phantom. The interobserver agreement for line pair reading was *κ* = 0.305, which implied fair agreement [15].

For the low contrast detectability of the contrast detail depth in Figure 3(b), observing the holes with smaller depths on the images implied the better image quality. For the chest, BodiiRay provided the average depth of 0.0363 inch, which was better than 0.0443 inch of Fujifilm FDR Go and 0.0397 inch of the Siemen Ysio Max. For the abdomen, BodiiRay and Siemens Ysio Max provided an equal average depth of 0.0350 inch and were better than 0.0443 inch of the Fujifilm FDR Go. For the extremity, BodiiRay and Siemens Ysio Max gave an equal average depth of 0.0163 inch, and it was better than 0.0180 inch of the Fujifilm FDR Go. However, the differences among the other system performance were only one or two steps apart. Notice that the results for the extremity setting were better than other settings. This was also from the lower the scattering of the extremity setting of the survey phantom. The interobserver agreement for the contrast detail depth reading was *κ* = 0.322, which implied fair agreement.

For the low contrast detectability of the contrast detail diameter in Figure 3(c), observing the holes with smaller diameters on the images meant better image quality. For the chest, BodiiRay provided the average of 0.093 inch and was better than 0.100 inch of Fujifilm FDR Go and 0.110 inch of the Siemens Ysio Max. For the abdomen, Siemens Ysio Max provided the average of 0.093 inch, which was better than 0.100 inch of BodiiRay and Fujifilm FDR Go. For the extremity, all results were equal to 0.08 inch. The interobserver agreement for the contrast detail diameter reading was *κ* = 0.513, which implied moderate agreement.

For the number of distinguished shades of the step wedge Figure 3(d), the results showed not much difference. BodiiRay and Siemens Ysio Max provided the average of 11.0 steps, which is the maximum number of the steps, for all anatomies. These were slightly better than Fujifilm FDR Go provided the averages of 10.7, 10.3, and 9.7 steps for the chest, the abdomen, and the extremity, respectively. The interobserver agreement for the step wedge reading was *κ* = 0.382, which implied fair agreement.

The means of the test object reading results for each DR system are shown in Table 2. Note that these means were calculated across the difference exposure settings to show the overall performance of each DR systems. The mean differences between the reading results of BodiiRay and Fujifilm FDR Go and between those of BodiiRay and Siemens Ysio Max are shown in Table 3. These mean differences were analyzed using one-tailed paired *t*-test, and their *p* values are also reported in Table 3. For high contrast resolution, BodiiRay provided the higher (better) mean of the line pair than Siemens Ysio Max at 0.133 lp/mm (*p* = 0.018), while BodiiRay and Fujifilm FDR Go provided no difference in the mean. For the low contrast detectability of the cylindrical hole depth, BodiiRay provided lower (better) mean of the depth of the cylindrical holes than Fujifilm FDR Go at 0.633 × 10^−2^ inches (*p* = 0.033), and BodiiRay also provided better mean of the depth than Siemens Ysio Max at 0.111 × 10^−2^ inches (*p* = 0.339). For the low contrast detectability of the cylindrical hole diameter, BodiiRay provided lower (better) mean of the diameter of the cylindrical holes than Fujifilm FDR Go at 0.222 × 10^−2^ inches (*p* = 0.173), and BodiiRay also provided lower (better) mean of the diameter than Siemens Ysio Max at 0.333 × 10^−2^ inches (*p* = 0.304). Finally, for the gray scale discrimination of the step wedge, BodiiRay provided better (higher) mean of the number of separable steps than Fujifilm FDR Go at 0.778 steps (*p* = 0.004), while BodiiRay and Siemens Ysio Max provided no difference in the mean. According to these results, BodiiRay provided slightly better results in all test object readings than the other systems, except for the equal means in the line pair readings between BodiiRay and Fujifilm FDR Go and in the step wedge readings between BodiiRay and Siemens YSIO Max. Some of these results showed statistical significance (*p* < 0.05), i.e., the contrast detail depth and the step wedge readings between BodiiRay and Fujifilm FDR Go, and the line pair readings between BodiiRay and Siemens YSIO Max.

**Figure 4:**
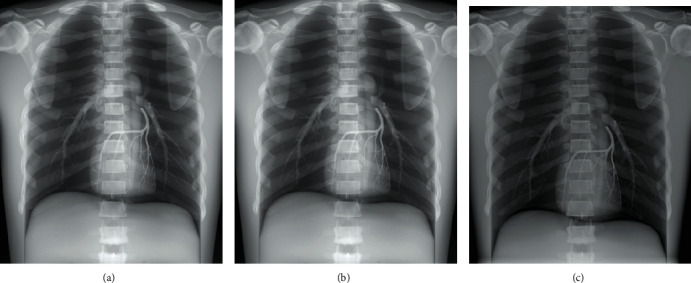
Radiographic images of the chest phantom from (a) BodiiRay, (b) Fujifilm FDR Go, and (c) Siemens Ysio Max.

**Figure 5:**
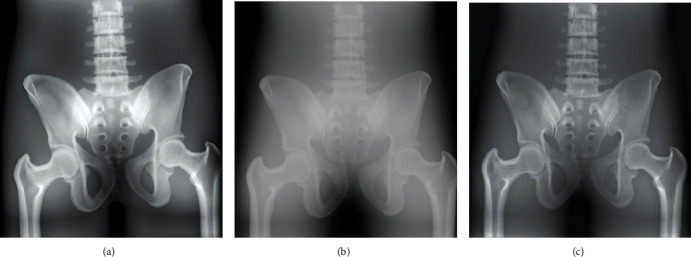
Radiographic images of the pelvis phantom from (a) BodiiRay, (b) Fujifilm FDR Go, and (c) Siemens Ysio Max.

**Figure 6:**
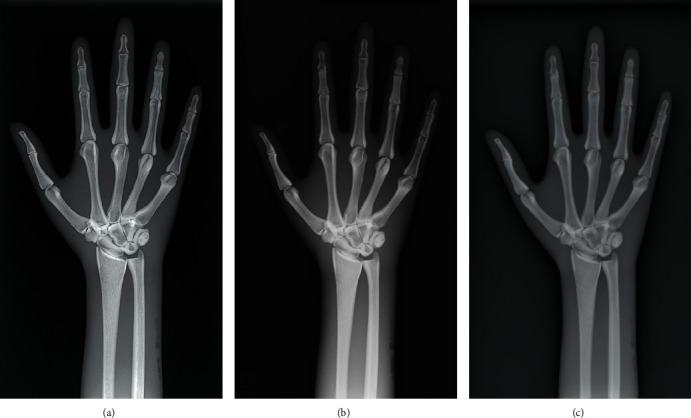
Radiographic images of the right hand phantom from (a) BodiiRay, (b) Fujifilm FDR Go, and (c) Siemens Ysio Max.

The radiographic images of three anthropomorphic phantoms of the chest, the pelvis, and the right hand, from BodiiRay, Fujifilm FDR Go, and Siemens Ysio Max, are shown in Figures 4–6, respectively. The contrasts of these images were set to their window centers and window widths suggested by those DR systems. For the chest phantom images in Figure 4, in general, the chest images from all DR systems provided good anatomy visibility, e.g., the visibility of the lungs, the heart, the bronchi, the spine, the bone, and the bone trabecular pattern. As can be seen, the image brightness of BodiiRay was between the image brightness of Fujifilm FDR Go and Siemens Ysio Max. The area of the spine superimposed with the diaphragm was clearly seen on the images of BodiiRay and Siemens Ysio Max. However, it was invisible on the image of Fujifilm FDR Go without brightness and contrast adjustment.

For the pelvis phantom images in Figure 5, BodiiRay image provided brighter pixels in the bone area than the others, especially in the thicker part of the bone. The bone trabecular pattern was visible. It also provided darker pixels in other tissue area than those from the other DR systems. In contrast, the Fujifilm FDR Go image seemed to be foggy in all bone and tissue areas. Although the bone trabecular pattern was still visible without brightness/contrast adjustment, the image from Siemens Ysio Max was similar to that from BodiiRay except that the bone trabecular area of BodiiRay showed more contrast.

For the hand phantom images in Figure 6, all DR systems provided similar image appearance. The bone structures, the bone trabecular pattern, and the tissue around the bone structures were clearly visible. The image brightness of Fujifilm FDR Go was higher than the others. The bone trabecular pattern of the BodiiRay image had higher contrast than the others.

Note that these images were from anthropomorphic phantoms and might not represent the radiographic image properties from the real human body part.

### 3.2. Volunteer Image Results

**Figure 7:**
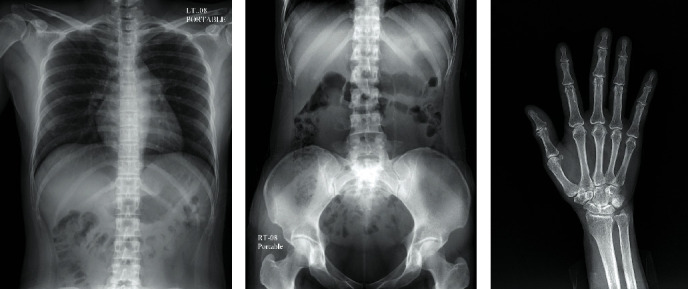
Sample radiographic images of the chest, the abdomen, and the right hand of the volunteers from the BodiiRay system.

**Figure 8:**
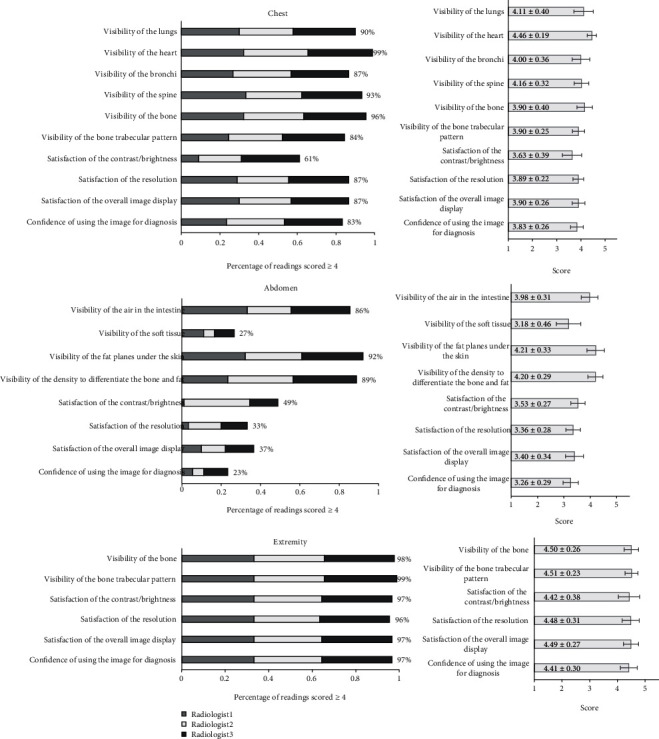
The bar graphs of the percentage of readings scored ≥4 (of 5-point scale) (left subplots) and average scores with error bars of their standard deviation (SD) and data labels of the mean ± SD inside the bars (right subplots) for the images of the chest, the abdomen, and the extremity. The percentage bars also showed the contribution portions from radiologists 1, 2, and 3 in the bars (using gray, white, and black colors, respectively).

Samples of the radiologic images of the chest, the abdomen, and the right hand of the volunteers are shown in Figure 7, and the evaluation results from the radiologists are shown in Figure 8. As aforementioned, the results were analyzed in the percentage of the readings that scored ≥4 (from a 5-point scale questionnaires). The criteria to be accepted as a good image quality, satisfaction, or confidence were set to be as more than 80% of the readings that scored ≥4. In Figure 8, the left subplots showed bar graphs of the percentage of the readings that scored ≥4 and the contribution of each radiologist in this percentage. The percentage indicated by the total length of the bar and the contribution portions from radiologists 1, 2, and 3 indicated by the bar sections colored using gray, white, and black, respectively. The right subplots showed bar graphs of the average scores with standard deviation error bars. The data labels inside the bars showed the mean ± standard deviation of the scores.

The number of the chest images from BodiiRay scored ≥4 was above 84% in terms of the visibility of the lungs, the heart, the bronchi, the spine, the bone, and the bone trabecular pattern, as well as in terms of the satisfaction of the image resolution and the overall image display. However, there was a limitation of the satisfaction on the image contrast/brightness, i.e., only 66% of the images scored ≥4, and the average score was 3.63 ± 0.39 of the standard deviation (SD). For the confidence of using the images for diagnosis, the number of the images scored ≥4 was above 83%.

The number of the abdomen images from BodiiRay scored ≥4 was above 86% in 3 categories, i.e., the visibility of the air inside the intestine, the visibility of the fat planes under the skin, and the visibility of the density to differentiate the bone and fat. However, there was a limitation of the visibility of the soft tissue, i.e., only 27% of the readings that scored ≥4, and the average score was 3.18 ± 0.46 SD. There were also limitations of the satisfaction in terms of the image contrast/brightness, the image resolution, and the overall image display, i.e., only 49%, 33%, and 37%, of the readings that scored ≥4, respectively, and the average scores were 3.53 ± 0.27 SD, 3.36 ± 0.28 SD, and 3.40 ± 0.34 SD, respectively. For the confidence of using the image for diagnosis, the number of the images scored ≥4 was only 23% and the average score of 3.26 ± 0.29 SD.

The number of the extremity images from BodiiRay scored ≥4 was above 96% and the average scores more than 4.42 in all categories, i.e., the visibility of the bone and the bone trabecular pattern, as well as the satisfaction of the image brightness/contrast, the image resolution, and the overall image display. Moreover, for the confidence of using the image for diagnosis, the number of the images scored ≥4 was above 97%, and the average score was 4.41 ± 0.30 SD.

## 4. Discussion

The results of the phantom images show that the images from BodiiRay are comparable to those from Fujifilm FDR Go and Siemens Ysio Max. This comparison is based on radiographic exposure settings generally used for a portable X-ray machine, e.g., without antiscatter grid, without perfect alignment between the X-ray source and the detector, and with low kV and low dose, and therefore, it might not represent the images from other settings. The images from Fujifilm FDR Go and Siemens Ysio Max are not at their best representation since the settings were fixed for the comparison. The purpose of this phantom image comparison is to gain a better understanding of the BodiiRay imaging algorithm and to prepare it for clinical evaluation.

The results from clinical evaluation of volunteer images show that BodiiRay providing sufficiently good image quality for using as radiographs of the chest, the abdomen, and the extremity. There are limitations in the image brightness/contrast, the image resolution, and overall image display to be improved for better digital radiographs. It needs to be noted that the clinical evaluation is based on three radiologists in one hospital. Therefore, it might not be generalized enough for other radiologists. The familiarity of a particular machine might affect reading ability [16] since different machines use different proprietary image processing algorithms and might be different from the reader's background.

The image results of the abdomen seem to be inferior to those of the chest and the extremity. This is due to the imaging challenges, e.g., the larger thickness of the abdomen, the variety of tissue types, the low dose radiographic exposure setting, and the lag of antiscatter grid. Other DR systems may suffer from this imaging challenge as well. Nowadays, many latest DR systems come with scatter correction software [17–20] that lower the dose and improve the image quality for this situation. The scatter correction software of BodiiRay [21] is forthcoming and will be soon available for upgrading.

## 5. Conclusions

The phantom results showed that the BodiiRay DR system provided image qualities comparable to two commercial DR systems, i.e., the Fujifilm FDR Go and Siemens Ysio Max, in terms of the high-contrast image resolution, the low-contrast image detectability, and the ability to differentiate grayscale levels. The volunteer images evaluated by three specialized radiologists demonstrated that the BodiiRay DR system provide sufficient image qualities for digital radiography of the chest, the abdomen, and the extremity.

## Figures and Tables

**Table 1:** The hardware specifications of the DR systems, BodiiRay, Fujifilm FDR Go DR-ID-1211SE, and Siemens Ysio Max Wi-D.

Specification	BodiiRay	Fujifilm FDR Go	Siemens Ysio Max
Tube voltage (kV)	40–125	40–133	40–150
Tube current max (mA)	400	400	540
Generator power (kW)	32	32	80
Focal spot (mm)	0.6/1.5	0.7/1.3	0.6/1.0
Target angle (degree)	14	16	12
Detector type	CsI, a-Si TFT	CsI, a-Si TFT	CsI, a-Si TFT
Detector size (inch)	14 × 17	14 × 17	14 × 17
Detector pitch (*μ*m)	139	150	148
Active matrix (pixel)	2476 × 3072	2336 × 2836	2354 × 2872
DQE at 1 lp/mm	44%	54%	50%
MTF at 1 lp/mm	57%	80%	61%

**Table 2:** The means of the test object reading results for each DR system averaged across the exposure settings.

Test objects	BodiiRay	Fujifilm FDR Go	Siemens YSIO Max
Line pair (lp/mm)	3.033	3.033	2.900
Contrast detail depth (inch)	0.029	0.036	0.030
Contrast detail diameter (inch)	0.091	0.093	0.094
Step wedge (step)	11.000	10.222	11.000

**Table 3:** The mean differences of the test object reading results and their *p* values compared between BodiiRay and Fujifilm FDR Go and between BodiiRay and Siemens YSIO Max.

Test objects	BodiiRay–Fujifilm FDR Go		BodiiRay–Siemens YSIO Max	
	Difference	*p* value	Difference	*p* value
Line pair (lp/mm)	0	0.500	0.133	0.018
Contrast detail depth (inch)	−0.633 × 10^−2^	0.033	−0.111 × 10^−2^	0.339
Contrast detail diameter (inch)	−0.222 × 10^−2^	0.173	−0.333 × 10^−2^	0.304
Step wedge (step)	0.778	0.004	0	NA^∗^

^∗^Unable to calculate since all paired data were equal.

## Data Availability

The image data used to support the findings of this study are available from the corresponding author upon request.
